# Informing Species Conservation at Multiple Scales Using Data Collected for Marine Mammal Stock Assessments

**DOI:** 10.1371/journal.pone.0017993

**Published:** 2011-03-28

**Authors:** Alana Grech, James Sheppard, Helene Marsh

**Affiliations:** 1 Centre of Excellence for Coral Reef Studies, James Cook University, Townsville, Queensland, Australia; 2 School of Earth and Environmental Sciences, James Cook University, Townsville, Queensland, Australia; 3 San Diego Zoo Institute for Conservation Research, San Diego Zoo, Escondido, California, United States of America; University of Glamorgan, United Kingdom

## Abstract

**Background:**

Conservation planning and the design of marine protected areas (MPAs) requires spatially explicit information on the distribution of ecological features. Most species of marine mammals range over large areas and across multiple planning regions. The spatial distributions of marine mammals are difficult to predict using habitat modelling at ecological scales because of insufficient understanding of their habitat needs, however, relevant information may be available from surveys conducted to inform mandatory stock assessments.

**Methodology and Results:**

We use a 20-year time series of systematic aerial surveys of dugong (*Dugong dugong*) abundance to create spatially-explicit models of dugong distribution and relative density at the scale of the coastal waters of northeast Australia (∼136,000 km^2^). We interpolated the corrected data at the scale of 2 km * 2 km planning units using geostatistics. Planning units were classified as low, medium, high and very high dugong density on the basis of the relative density of dugongs estimated from the models and a frequency analysis. Torres Strait was identified as the most significant dugong habitat in northeast Australia and the most globally significant habitat known for any member of the Order Sirenia. The models are used by local, State and Federal agencies to inform management decisions related to the Indigenous harvest of dugongs, gill-net fisheries and Australia's National Representative System of Marine Protected Areas.

**Conclusion/Significance:**

In this paper we demonstrate that spatially-explicit population models add value to data collected for stock assessments, provide a robust alternative to predictive habitat distribution models, and inform species conservation at multiple scales.

## Introduction

The data that inform conservation planning and the design of marine protected areas (MPAs) are primarily spatially explicit [1],[2]. Spatial information that represents ecological features needs to: (1) extend across the entire planning region; and, (2) match the scales of population biology and dispersal ability of target species [3]. In the marine environment, the size of planning regions can vary from local scales such as small bays and estuaries (e.g. Monterey Bay, California 650 km^2^) to regional scales such as large networks of marine reserves (e.g. Papahānaumokuākea Marine National Monument, Hawaii 360,000 km^2^). Ecological scales can vary from 10 s of km^2^ for isolated, sedentary species with small geographic ranges (e.g. Banggai cardinalfish), to 100,000 s of km^2^ for migratory species (e.g. marine turtles, tuna, some species of sharks and large whales). The scales of planning regions and ecological features are rarely congruent [4], presenting a major constraint to the effective management of marine species [5].

Marine mammals are some of the most highly dispersed species with geographic ranges up to 300,000,000 km^2^ [6]. Spatial information on the distribution of marine mammals at ecological scales is typically difficult and costly to obtain. Furthermore, the lack of spatially-explicit environmental and sighting data precludes the use of habitat suitability modelling [7] to predict the distribution of most marine mammal species at broad spatial scales [8]. Most research is limited to predicting the distribution of marine mammals within a small proportion of their range (mainly known feeding or calving areas e.g. [9]–[12]). The outputs of fine-scale models of species distribution are relevant to species conservation at local scales and within small planning regions, however, they do not inform the management of marine mammals at regional scales or across their broader distributional ranges.

Dugongs (*Dugong dugon*) occur in the shallow, protected coastal waters of some 40 countries and territories in the tropical and subtropical Indo-West Pacific. As the only herbivorous mammal that is strictly marine, dugongs are often used as a flagship species because of their high biodiversity and cultural values. Although dugongs are seagrass community specialists, their habitat needs are not yet sufficiently understood to predict their distribution at broad spatial scales using habitat modelling [13]. Dugongs do not exploit all of the available food resources within the seagrass pastures in their range. Instead, dugongs select habitats based on multiple environmental and nutritional factors including bathymetry, seagrass species, and seagrass biomass, starch and nitrogen content [14]–[17].

**Figure 1:**
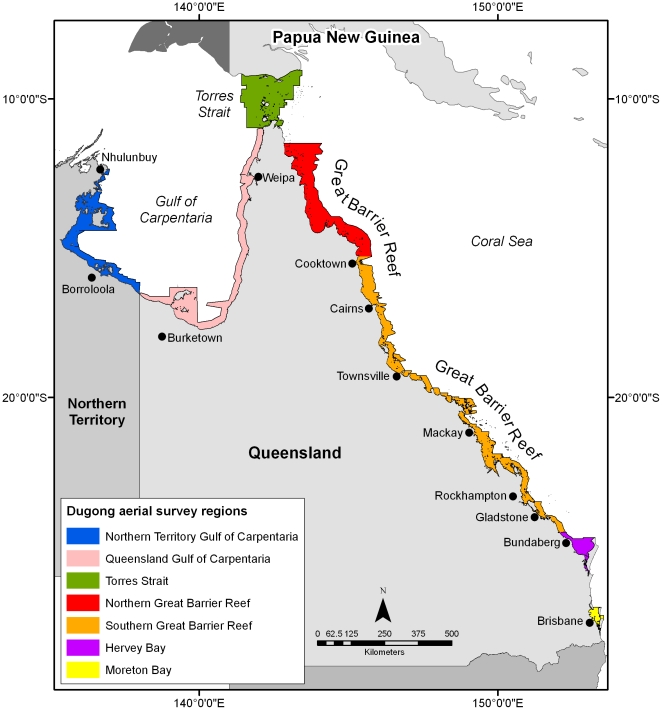
The seven dugong aerial survey regions of northeast Australia. Systematic aerial surveys have been used to monitor the abundance and distribution of dugong populations in northeast Australia since the mid 1980s using strip transect methodology [20]. The outputs of the aerial surveys were used in this paper to develop spatially-explicit models of dugong distribution and relative density in each of the seven survey regions.

Dugongs are of high cultural and nutritional value to Indigenous Australians and northern Australia is internationally recognised as supporting the most globally significant remaining dugong populations [18], [19]. Based on the length of the coastline, around a quarter of the dugong's range occurs in northern Australia between Moreton Bay in Queensland (Figure 1) and Shark Bay in Western Australia. Consequently, dugong conservation is a high priority in northern Australia. A predictive habitat distribution model for dugongs at the scale of northern Australia (>100,000 km^2^) would require information on the distribution of: (1) seagrass habitat community composition; and (2) the various factors that influence the choice of seagrass species or habitats by dugongs. This information is currently unavailable for most of the habitats exploited by dugongs in northern Australia.

Systematic aerial surveys have been used to monitor the abundance and distribution of dugong populations in northeast Australia (Figure 1) since the mid 1980s using transect methodology [20]. These surveys were conducted for stock assessment purposes over ∼136,000 km^2^; almost half of their range in northern Australian waters from Moreton Bay in Queensland, the southern extremity of the dugong's range on the east coast (27°50′21″S), through the Gulf of Carpentaria (12°13′8″S) (Figure 1). The surveyed area is substantially more than their area of occupancy within the region. Grech and Marsh (2007) [21] subsequently used the dugong abundance data collected from the aerial surveys in the Great Barrier Reef region (Figure 1) to develop spatially-explicit models of dugong distribution and relative density in the coastal waters of the region (∼73,000 km^2^). These models have informed dugong conservation initiatives within the Great Barrier Reef World Heritage Area because they effectively delineate the spatial distribution of dugongs at the required scale [22].

This paper updates and extends the spatially-explicit model of dugong distribution and relative density in the Great Barrier Reef region [21] to the entire coast of northeast Australia (Figure 1). We developed the models of dugong distribution and relative density using information collected from the 20-year time-series of dugong aerial surveys and geostatistics. We also demonstrate how data collected for stock assessments can be used to inform dugong conservation at multiple scales. The models add value to data collected for dugong stock assessments and provide a robust alternative to predictive habitat distribution models.

## Methods

### Data sets

Marsh's group undertook systematic aerial surveys of northeast Australia in seven survey regions (Figure 1) approximately every five years (Table 1) from 1985–2007 [20], [23]–[29] using the strip transect technique developed for environments with heterogeneous water visibility and described by Marsh and Sinclair (1989) [20] and Pollock et al. (2006) [30]. Pollock et al. (2006) [30] found that strip transects are more appropriate for estimating dugong abundance in heterogeneous environments than line transect methods. The survey regions were divided into blocks containing systematic transects of varying length. These transects were typically perpendicular to the coast across the depth gradient and 200 m wide at the water's surface on either side of the aircraft.

**Table 1:** Dugong aerial survey year and month^1^ for the seven survey regions (Figure 1).

Survey Year	Moreton Bay	Hervey Bay	Southern Great Barrier Reef	Northern Great Barrier Reef	Torres Strait	QLD Gulf of Carpent-aria	NT Gulf of Carpent-aria
1985				Apr* Nov*			
1986			Sep* Nov*				
1987			Sep*		Nov		
1988		Aug					
1990				Nov			
1991					Nov	Dec*	
1992		Nov	Nov				
1993		Dec					
1994		Nov	Nov		Dec*	Dec*	Nov
1995				Nov			
1996					Nov		
1997						Dec	
1999	Oct*		Oct				
2000	Dec						
2001	Apr Nov Dec	Apr Nov			Nov		
2005	Nov	Nov	Nov		Nov*		
2006		Nov*		Nov	Nov	Nov*	
2007						Nov	Nov

Multiple surveys were conducted in the same survey year where there is more than one month identified in the same cell. No aerial surveys were conducted in 1989, 1998, 2002, 2003 and 2004.

*denotes partial aerial surveys of the region.

1 April (Apr), September (Sep), October (Oct), November (Nov) and December (Dec).

Using the technique of Grech and Marsh (2007) [21], we developed spatially-explicit models of dugong distribution and relative density using information from Moreton Bay (6 surveys), Hervey Bay (8), the southern Great Barrier Reef region (7), northern Great Barrier Reef (5), Torres Strait (7), Queensland Gulf of Carpentaria (5) and Northern Territory Gulf of Carpentaria (2) (Table 1; Figure 1). By combining data collected over more than 20 years, the models should account for temporal changes in the use of various regions by dugongs including movements resulting from events such as seagrass dieback during cyclone and flood events [31], [32].

Most aerial surveys were conducted in late spring or early summer when weather and sea states provide optimum survey conditions (Table 1). In higher latitudes such as Moreton Bay and Hervey Bay in southeast Queensland (Figure 1), dugongs move in response to low water temperatures in winter [33], [34]. Aerial surveys were conducted during summer and winter in both Moreton Bay and Hervey Bay to account for these seasonal differences.

### Data analysis

All the aerial surveys estimated absolute dugong abundance by correcting sightings for perception bias (animals that are available to, but missed by, observers) and availability bias (animals that are unavailable to observers because of water turbidity) *sensu* Marsh and Sinclair (1989) [20]. Prior to the development of the methodology of Pollock et al. (2006) [30], corrections for these biases were applied at the spatial scale of entire surveys (>1,000 km^2^) making them inappropriate to use in the spatially-explicit models which we developed at the scale of 2 km * 2 km planning units. Thus the models were based on relative rather than absolute population estimates, nonetheless, relative densities among regions should be approximately comparable [21].

We corrected the spatial data from the aerial surveys for differences in sampling intensity and area sampled between surveys using equations described in Grech and Marsh (2007) [21]. We investigated the spatial autocorrelation of the data by a variogram analysis using the Geostatistical Analyst extension of ArcGIS® 9.3 (Environmental Systems Research Institute 2009). We then interpolated the corrected data to the spatial extent of the aerial surveys (Figure 1) using the geostatistical estimation method of universal kriging and the Spatial Analyst© extension of ArcGIS® 9.3 (Environmental Systems Research Institute 2009).

As independent data on dugong abundance at the scale of northeast Australia do not exist, we used a re-substitution approach to validate the individual spatially-explicit population models [35], [36]. For each model, a random sub-sample of observations constituting 30% of the total observations were removed and then tested against dugong distribution and relative density predicted from the krige using the remaining 70% of observations.

We estimated dugong distribution and relative density at a planning unit of 2 km * 2 km because this scale: (1) corresponds with the scale of the aerial survey data allowing the model to account for: (a) slight changes in altitude of the aircraft (which affects transect width at the surface); and, (b) the blind area under the aircraft; and, (2) is recommended under Criterion B of the *International Union for Conservation of Nature and Natural Resources Red List* [37].

Density estimates are regarded as robust surrogates of habitat utilization [38]. We grouped our density estimates based on inspection of their frequency distributions as follows: low density areas had relative dugong densities of 0 dugongs/km^2^; medium density 0.0015<0.25 dugongs/km^2^; high density areas 0.25≤0.5 dugongs/km^2^; and very high density areas >0.5 dugongs/km^2^. We included planning units with 0 dugongs/km^2^ to ensure that the spatial layers extended across the entire survey region (Figure 1) and because dugongs are likely to move across inshore units where they were not detected during the surveys [31], [34].

## Results

The average relative dugong density in the *entire* coast of northeast Australia covered by aerial surveys was 0.17 dugongs/km^2^ and ranged from 0 to 9.0 dugongs/km^2^ (Table 2). Density was highest in Torres Strait (mean  = 0.55 dugongs/km^2^), Hervey Bay (0.43 dugongs/km^2^), Moreton Bay (0.19 dugongs/km^2^) and the northern Great Barrier Reef region (0.16 dugongs/km^2^). The planning units with the highest relative densities were in Moreton Bay (9.0 dugongs/km^2^), Torres Strait (6.49 dugongs/km^2^), the northern Great Barrier Reef region (6.03 dugongs/km^2^) and Hervey Bay (4.56 dugongs/km^2^). The southern Great Barrier Reef region and coastal waters of the Gulf of Carpentaria had the lowest mean (<0.07 dugongs/km^2^) and maximum density estimates (<1.92 dugongs/km^2^).

**Table 2:** Mean, range and standard deviation of the relative density estimates (dugongs/km^2^) within the seven survey regions.

Survey region	Area (km^2^)	Mean	Range	Standard deviation
Moreton Bay	2,192	0.19	0–9.0	0.78
Hervey Bay	6,156	0.43	0–4.56	0.62
Southern Great Barrier Reef^1^	33,676	0.02	0–1.92	0.07
Northern Great Barrier Reef	20,132	0.16	0–6.03	0.39
Torres Strait	29,764	0.55	0–6.49	0.67
Gulf of Carpentaria (QLD)	34,484	0.05	0–0.92	0.11
Gulf of Carpentaria (NT)	26,184	0.07	0–1.10	0.11
*Northeast Australia*	*152,588*	*0.17*	*0–9.0*	*0.42*

**Figure 2:**
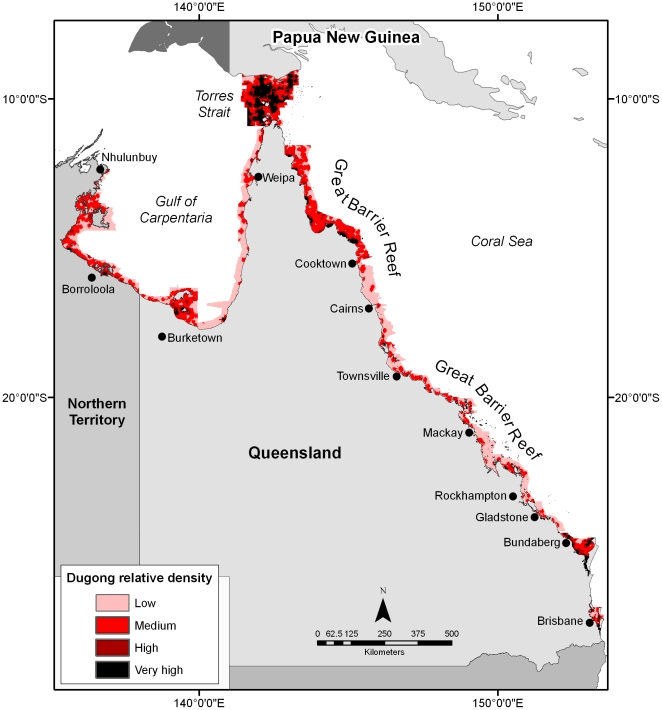
Spatially-explicit population models of dugong distribution and relative density in northeast Australia. The spatially-explicit models were interpolated from a 20-year time series of systematic aerial surveys of dugongs at the scale of 2 km * 2 km planning units. Planning units were classified as low, medium, high and very high dugong density on the basis of the relative density of dugongs estimated from the models and a frequency analysis. The model of dugong distribution and relative density in the southern Great Barrier Reef region is from Grech and Marsh (2007) [21].

Planning units of very high and high relative dugong density in Moreton Bay and Hervey Bay were adjacent to the mainland coast and islands (Figure 2; Figure S1). In the southern Great Barrier Reef region, planning units of very high relative density were north of Hinchinbrook Island, and in Cleveland Bay, Shoalwater Bay and Port Clinton (Figure 2; Figure S2). In the northern Great Barrier Reef region, the highest density planning units were adjacent to Friendly Point and Port Stewart and between Lookout Point and Princess Charlotte Bay (Figure 2; Figure S3). In Torres Strait, planning units of very high and high relatively density occurred throughout the survey region (∼30,000 km^2^; Figure 2; Figure S3). In the Gulf of Carpentaria, planning units of very high relative density were northwest of Normanton, and south of the Wellesley Islands and the Sir Edward Pellew Group (Figure 2; Figure S4). The planning units that we identified as very high dugong density areas relative to other units were consistent with the regions identified as important habitats for dugongs in northeast Australia by [27]–[29]. However, our methodology facilitates quantitative spatial comparisons across regions for species conservation and the design of MPAs at a national scale.

The total area of dugong planning units in northeast Australia predicted to be of very high, relative density was 15,332 km^2^; high: 12,432 km^2^, medium: 63,024 km^2^ and low: 61,800 km^2^ (Table 3; Figure 2). Torres Strait (Figure S3) and Hervey Bay (Figure S1) had the greatest proportion of planning units of very high and high dugong relative density within their survey regions (Table 3). The southern Great Barrier Reef region (2) and Gulf of Carpentaria waters in Queensland (Figure S4) had the lowest proportion of planning units of very high and high dugong density within their survey regions (Table 3).

**Table 3:** Total area (km^2^) and proportion (%) of dugong planning units of low, medium, high and very high relative densities within the seven survey regions.

	Dugong relative density			
Survey region	*Low*	*Medium*	*High*	*Very high*
Moreton Bay	868 (39.6)	1,084 (49.5)	112 (5.1)	128 (5.8)
Hervey Bay	492 (8.0)	2,340 (38.0)	1,412 (22.9)	1,912 (31.1)
Southern Great Barrier Reef^1^	22,724 (67.5)	10,496 (31.2)	316 (0.9)	140 (0.4)
Northern Great Barrier Reef	3,436 (17.1)	13,684 (68.0)	1,540 (7.6)	1,472 (7.3)
Torres Strait	2,416 (8.1)	10,504 (35.3)	5,944 (20.0)	10,900 (36.6)
Gulf of Carpentaria (QLD)	20,528 (59.5)	11,996 (34.8)	1,496 (4.3)	464 (1.3)
Gulf of Carpentaria (NT)	11,336 (43.3)	12,920 (49.3)	1,612 (6.2)	316 (1.2)
*Northeast Australia*	*61,800 (40.5)*	*63,024 (41.3)*	*12,432 (8.2)*	*15,332 (10.0)*

## Discussion

We enabled the 20-year time series of data collected for dugong stock assessments in northeast Australia to be used for species conservation and the design of MPAs at local, regional and national scales by developing spatially-explicit models of dugong distribution and relative density (Figure 2). Torres Strait (Figure S3) was identified as the most significant dugong habitat in northeast Australia and the most globally significant known habitat for any member of the Order Sirenia. Hervey Bay and Moreton Bay (Figure S1); Hinchinbrook Island, Cleveland Bay, Shoalwater Bay and Port Clinton (Figure S2); Friendly Point, Port Stewart and between Lookout Point and Princess Charlotte Bay (Figure S3); northwest of Normanton and south of the Wellesley Islands, and the Sir Edward Pellew Group (Figure S4) were identified as regionally important dugong habitats. The modelling also indicated that the dugong habitat in Torres Strait extended west of the survey region, prompting a vessel survey that led to discovery of the largest seagrass meadow yet mapped in Australian waters [39]. Until recently it was considered unsafe to conduct light-aircraft surveys in far western Torres Strait due to its distance (∼70–150 km) from the nearest mainland or islands. Our modelling has also catalysed funding for an 11,000 km^2^ aerial survey of this region. We will model the results of this survey using the approach described here and add the results to the existing layer of dugong distribution and relative density of northeast Australia.

Our approach makes the assumption that the model of dugong distribution and relative density developed from the time series of aerial surveys is a robust index of a region's conservation value for dugongs. This assumption is justified for most regions (especially remote areas) because: (1) specialised areas of high conservation value such as calving or mating areas and migratory corridors have not been identified; and (2) density estimates are regarded as robust surrogates of habitat utilization [38]. However, the model is likely to underestimate the historical density of dugongs along the urban coast of eastern Queensland (Figure 2). Marsh et al. (2005) [13] find that the number of dugongs in six locations along the urban coast declined dramatically between the 1960s and 1990s and that anthropogenic impacts may have reduced the region's carrying capacity for dugongs (e.g. [40]). It is impossible to estimate the historical spatial distribution of dugongs along the urban coast of Queensland as most of the decline occurred in the 1960s or 1970s, before the implementation of aerial surveys and systematic monitoring of seagrass habitats [41]. However, this lack should not increase the uncertainty in the application of the models of dugong distribution and relative density for two reasons: (1) the spatial scale of dugong management in northeast Australia is far broader than any reduction in the area used by dugongs within their range; and, (2) the models are used to inform current management actions rather than past management failures.

### Ecological insights

The spatially-explicit models suggest that the broad-scale patterns of dugong distribution in coastal regions of northeast Australia are determined by the physical characteristics of their seagrass habitats: exposure to wind and wave activity, tidal ranges and seabed current stress [42], [43]. Examples of very high and high dugong density areas in protected waters include: (1) the continental shelf of western Torres Strait; shallow, north-facing bays of southeast Queensland; and, (3) the protected shallow coastal waters protected surrounding the Wellesley Islands and Sir Edward Pellew Group of the Gulf of Carpentaria (Figure 2). Conversely, regions of low dugong density included the exposed east-facing coastlines of southeast Queensland and west-facing coastlines of the Gulf of Carpentaria (Figure 2). The spatial models also indicate that currently dugongs do not exploit all available seagrass meadows. For example, Trinity Inlet, an area adjacent to Cairns in northeast Australia (Figure S2), had a low dugong density even though the region supports extensive seagrass habitats [43]. Whether this is a result of this habitat being unsuitable for dugongs or local depletion is not known.

The broad-scale patterns of dugong distribution predicted by our model can assist in the identification of important dugong habitats in data-poor areas of the Indo-Pacific. It is likely that dugongs exhibit habitat preferences similar to those in northeast Australia throughout their range (i.e. shallow (>−30 m), coastal waters, bays and estuaries with low wave exposure [43]). The continental shelf of western Torres Strait (a land bridge that linked Australia and Papua New Guinea ∼10,000 years ago) supported the greatest proportion of very high and high dugong density areas; regions of similar geological history may also have been important dugong habitats. For example, Palk Strait, site of the land bridge between India and Sri Lanka used to be significant dugong habitat [44] but anecdotal information suggests that dugong numbers in the area are now seriously depleted [45].

### Informing species conservation across multiple scales

Australia aims to realise its international commitments as a signatory to the Convention on Biological Diversity through the significant expansion of its existing Marine Protected Area network throughout Australia's Exclusive Economic Zone by 2012. The central component of Australia's Oceans Policy (Commonwealth of Australia 1998) is the development of Marine Bioregional Plans and a National Representative System of Marine Protected Areas in Commonwealth (Australian) waters. Australia's Commonwealth (Federal), State and Northern Territory governments are working together to implement this initiative. The models of dugong distribution and relative density currently inform Australia's Oceans Policy and species conservation initiatives of local and State (Queensland and Northern Territory) governments across multiple scales. In the following section, we provide specific examples of the application of the models at local, regional and national scales to demonstrate the merits of using survey data collected for stock assessment in species conservation and the design of MPAs.

#### Local scales

One of the major outcomes of our modelling exercise was the improved understanding of the relative importance of the seven survey regions to dugong conservation in northeast Australia. Torres Strait has the greatest number of very high dugong density planning units when compared to the other survey regions of northeast Australia (Figure S3; Table 3). The models have been provided to the Torres Strait Regional Authority and Indigenous communities within the region to inform dugong management at local scales (<100 km^2^). Primarily, the models assist with the development of management decisions related to the harvest of dugongs (including spatial closures) at the scale of Torres Strait (∼33,000 km^2^).

Hervey Bay also has a large proportion of very high and high dugong density planning units relative to its size (Figure S1; Table 3). Our model of dugong distribution and relative density in Hervey Bay directly informed the design of the network of marine reserves within the recently declared Great Sandy Marine Park (Queensland Department of Environment and Resource Management 2006).

#### Regional scales

Dugongs are listed as vulnerable to extinction under schedule 3 of the Queensland Nature Conservation (Wildlife) Regulation of 1994 and were one of several explicit reasons for the World Heritage listing of the Great Barrier Reef region [46]. The Australian and Queensland governments are using the spatially-explicit dugong population models of the southern and northern Great Barrier Reef (Figure 2) to inform fisheries management decisions [22] and to test the efficacy of the ecosystem-scale network of marine reserves within the Great Barrier Reef Marine Park [47], [48]. The models have been spatially analysed in conjunction with threat data to identify areas where dugongs are at risk of drowning in commercial gill-nets [49] and to rapidly assess the risk to dugongs from all of their known anthropogenic threats [50]. The outputs of Grech et al. (2008)[49] and Grech and Marsh (2008) [50] were featured in the Great Barrier Reef Marine Park Authority's Outlook Report (2009) [51] that summarised the past and present condition of the environmental values of the Great Barrier Reef and possible future scenarios for the region [22].

#### National scales

The Australian Government is using the models of dugong distribution and relative abundance at the scale of northeast Australia (Figure 2) to assist in developing Marine Bioregional Plans and the National Representative System of Marine Protected Areas. In addition, the Australian Government is using the models to assist in developing a Wildlife Conservation Plan for dugongs, which is designed to establish the research and management actions necessary to support the survival of dugong populations at the scale of northern Australia.

### Adding value to abundance surveys for stock assessment

Many government agencies have developed comprehensive and dedicated monitoring programmes to estimate the size and trends of marine mammal populations for stock assessment (e.g. National Oceanic and Atmospheric Administration and Fish and Wildlife Service in the US and the Australian Antarctic Division in Australia). For example, the US Marine Mammal Protection Act of 1972 and subsequent amendments mandates the use of the Potential Biological Removal technique to estimate the maximum number of animals that may be removed from a stock [52]. This technique requires the following information for stocks of conservation concern: estimates of the absolute abundance (which are very difficult to obtain because survey techniques rarely meet the underlying assumption of line transect surveys that all animals on the tract-line are detected) and life history parameters (which can also be difficult to estimate). Our approach, which has much less demanding information requirements, demonstrates that information collected from systematic surveys is valuable to species conservation even when the absolute population is unknown and/or the power of the surveys to detect trends is limited [53]. Nonetheless, the following conditions must be met if stock assessment data are to be used for spatially-explicit population modelling: (1) surveys need to collect spatial information and be designed systematically and conducted consistently over time; (2) surveys need to be performed over a long time period to capture the movement of the target species in response to habitat change; and (3) the spatial extent of surveys must cover a large proportion of the distributional range of the study species. We recommend wider application of data from abundance surveys of marine mammals that meet these criteria to develop spatially-explicit models that inform species conservation across multiple scales.

## Supporting Information

Figure S1 Spatially-explicit population models of dugong distribution and relative density in Moreton Bay and Hervey Bay.
(TIF)

Figure S2 Spatially-explicit population models of dugong distribution and relative density in the southern Great Barrier Reef.
(TIF)

Figure S3 Spatially-explicit population models of dugong distribution and relative density in the northern Great Barrier Reef and Torres Strait.
(TIF)

Figure S4 Spatially-explicit population models of dugong distribution and relative density in the Gulf of Carpentaria.
(TIF)
