# Supplementary figures and images for: Informing Species Conservation at Multiple Scales Using Data Collected for Marine Mammal Stock Assessments
Source: PLoS One. 2011 Mar 28;6(3):e17993. doi: 10.1371/journal.pone.0017993 (PMC3065465; doi:10.1371/journal.pone.0017993)

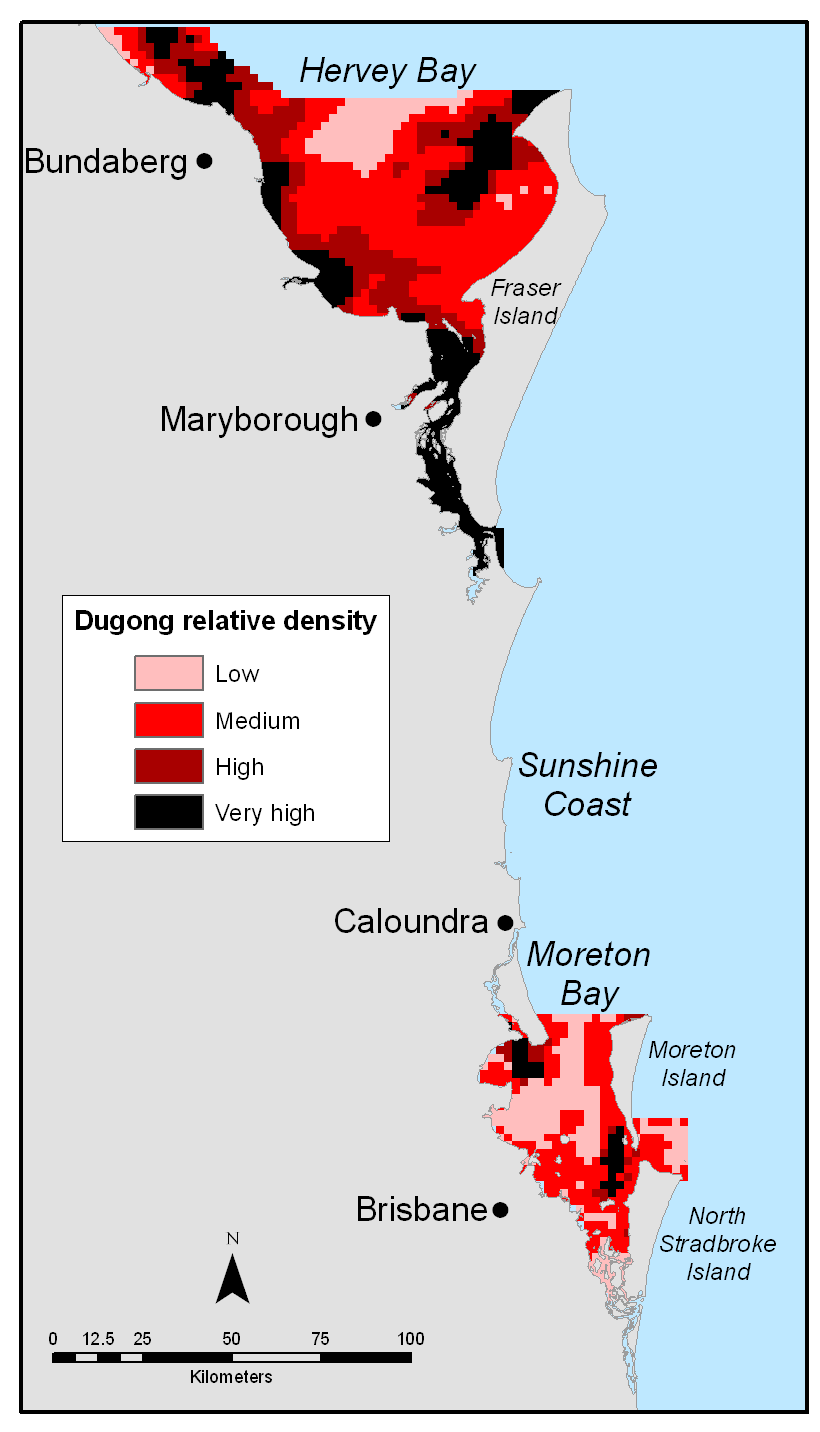

Supplement: Figure S1 — Spatially-explicit population models of dugong distribution and relative density in Moreton Bay and Hervey Bay. (TIF) [file pone.0017993.s001.tif]

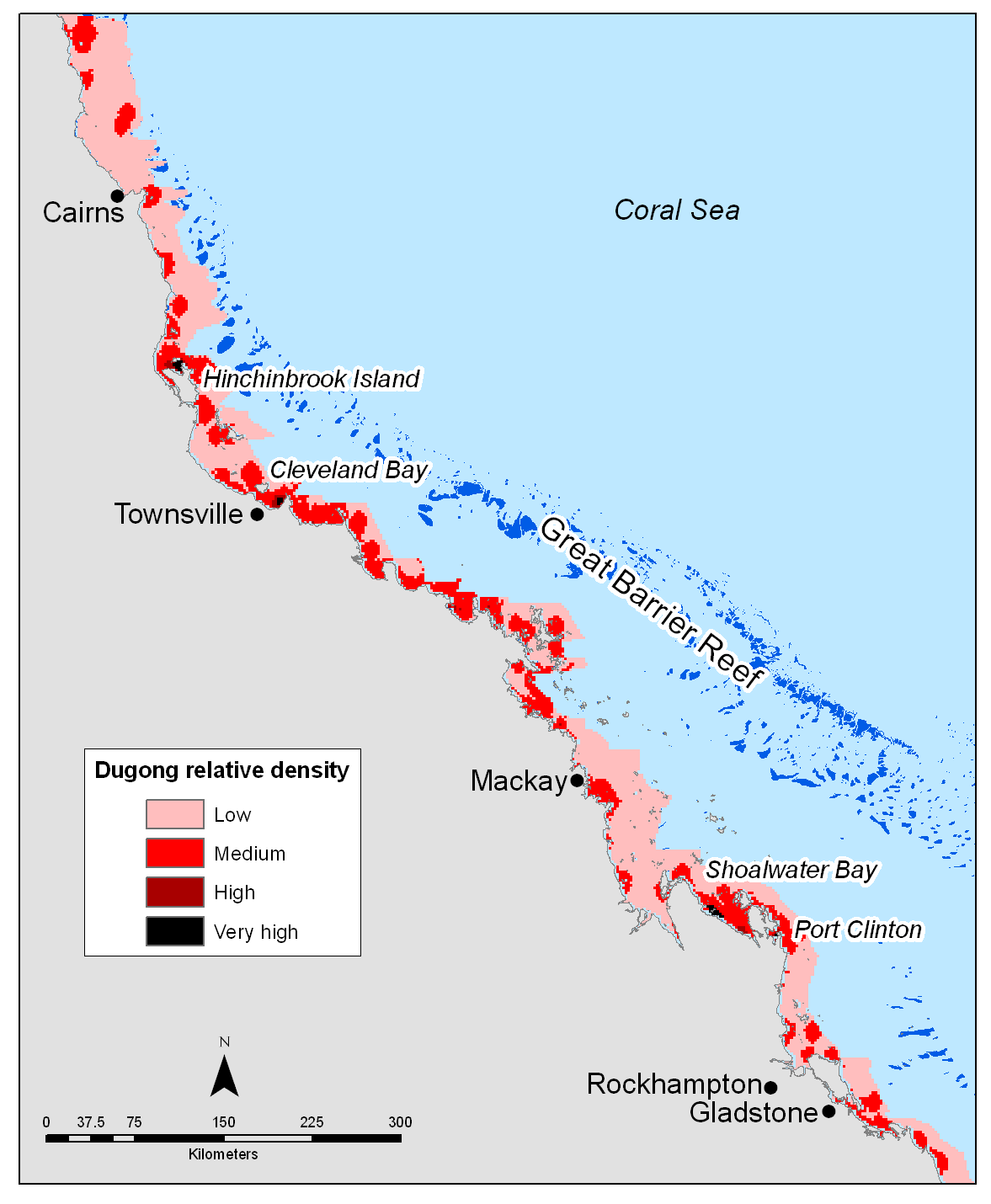

Supplement: Figure S2 — Spatially-explicit population models of dugong distribution and relative density in the southern Great Barrier Reef. (TIF) [file pone.0017993.s002.tif]

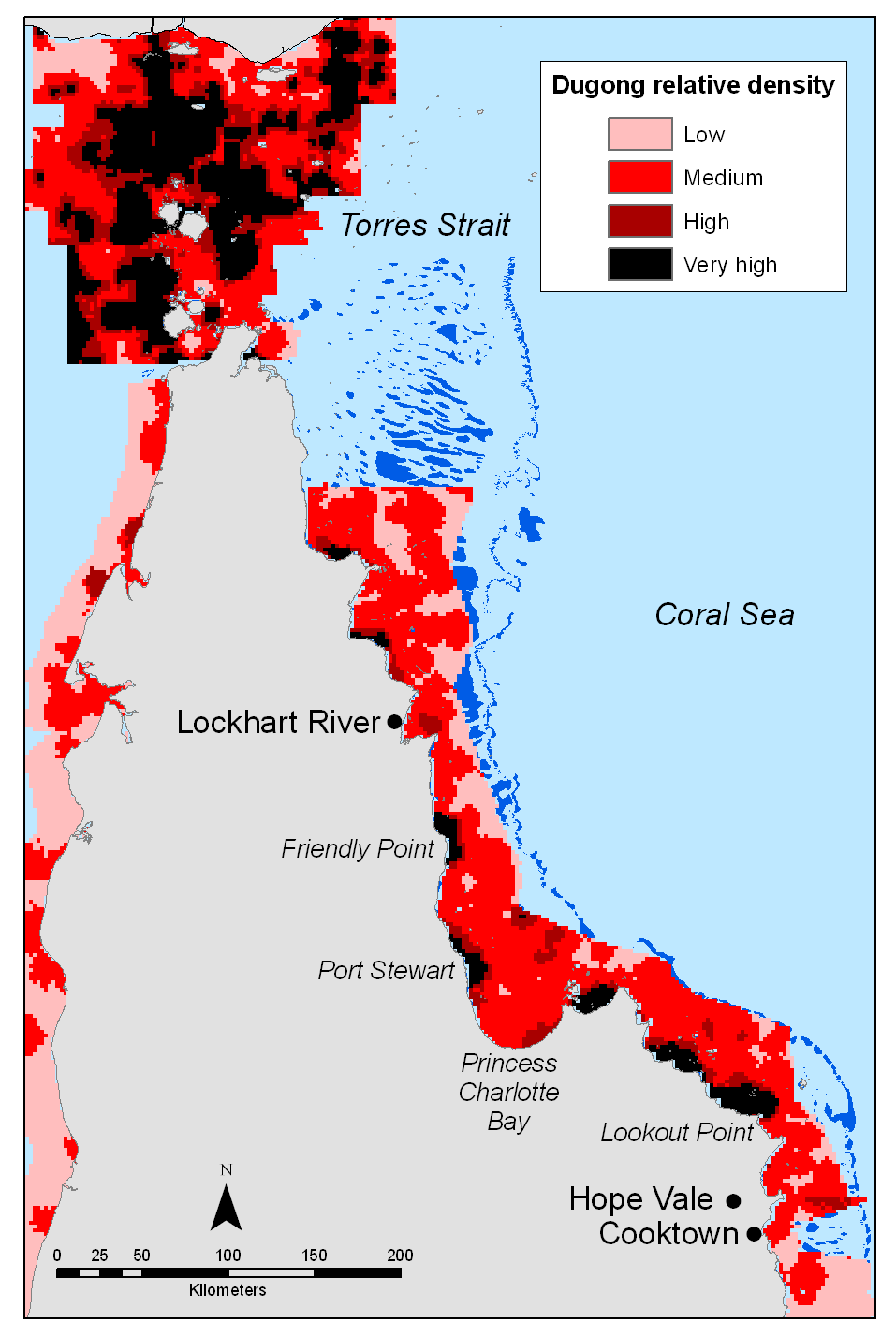

Supplement: Figure S3 — Spatially-explicit population models of dugong distribution and relative density in the northern Great Barrier Reef and Torres Strait. (TIF) [file pone.0017993.s003.tif]

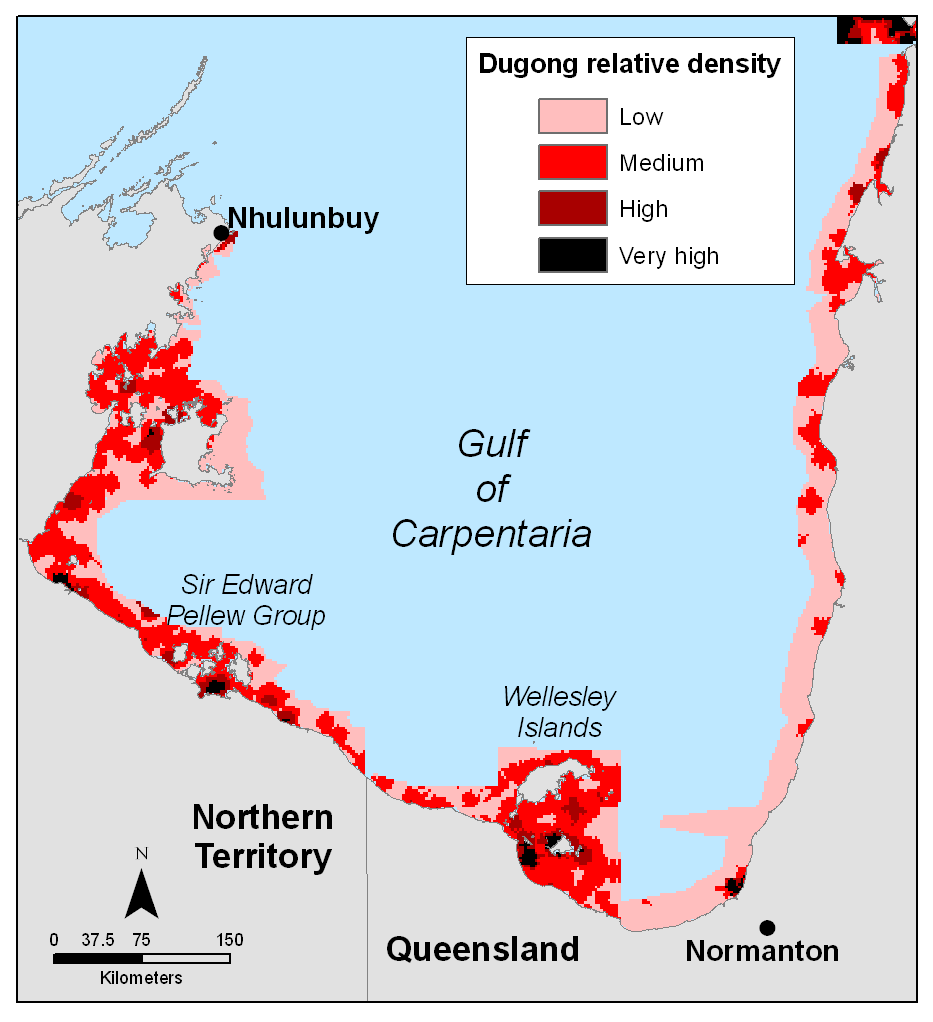

Supplement: Figure S4 — Spatially-explicit population models of dugong distribution and relative density in the Gulf of Carpentaria. (TIF) [file pone.0017993.s004.tif]
